# Supplementary material for: Spectrum of Genetic Mutations in Korean Pediatric Acute Lymphoblastic Leukemia
Source: J Clin Med. 2022 Oct 26;11(21):6298. doi: 10.3390/jcm11216298 (PMC9658397; doi:10.3390/jcm11216298)
Supplement: Supplementary file 1 [file jcm-11-06298-s001.zip › jcm-1985174-supplementary.pdf]

Supplementary Table S1. Gene targets of SM acute leukemia panel

**RAS pathway**	*KRAS, NF1, NRAS, FLT3, PTPN11*
**Receptor/kinase**	*FBXW7, JAK1, JAK2, JAK3, NOTCH1, NOTCH3*
**Tumor suppressor**	*CDKN2A, CDKN2B, PTEN, TP53, RB1*
**Transcription factor**	*CREBBP, ETV6, GATA1, GATA2, GATA3, IKZF1, MYC, NT5C2, SETD2*
**Others**	*ANKRD26, ASXL1, BCOR, BRAF, CALR, CBL, CHEK1, CSF3R, DDX41, DNMT3A, EGFR, EP300, ERG, EZH2, FAM5C, HNRNPK, IDH1, IDH2, IL7R, KIT, KMT2A, MPL, NPM1, PAX5, PDGFRA, PHF6, PRAME, RAD21, RUNX1, SETBP1, SF3B1, SH2B3, SMC1A, SMC3, SRSF2, STAG2, TERT, TET2, TPMT, U2AF1, WTI, ZRSR2*
